# Effect of Invasive Mechanical Ventilation at Birth on Lung Function Later in Childhood

**DOI:** 10.3389/fped.2022.912057

**Published:** 2022-06-30

**Authors:** Paola Di Filippo, Giulia Dodi, Sabrina Di Pillo, Francesco Chiarelli, Marina Attanasi

**Affiliations:** Pediatric Allergy and Respiratory Unit, Department of Pediatrics, University of Chieti, Chieti, Italy

**Keywords:** prematurity, mechanical ventilation, DLCO, surfactant, FeNO, newborn

## Abstract

**Background:**

Despite recent neonatal care improvements, mechanical ventilation still remains a major cause of lung injury and inflammation. There is growing literature on short- and long-term respiratory outcomes in infants born prematurely in the post-surfactant era, but the exclusive role of mechanical ventilation at birth in lung function impairment is still unclear. The aim of this study was to assess the effect of neonatal mechanical ventilation on lung function parameters in children born ≤ 32 weeks of gestational age at 11 years of age.

**Materials and Methods:**

In total, 55 ex-preterm children born between January 1, 2006 and December 31, 2007 were enrolled at 11 years of age. Neonatal information was obtained from medical records. Information about family and personal clinical history was collected by questionnaires. At 11 years of age, we measured spirometry parameters, lung volumes, diffusing lung capacity, and fractional exhaled nitric oxide. In addition, an allergy evaluation by skin prick test and eosinophil blood count were performed. A multivariable linear or logistic regression analysis was performed to examine the associations of mechanical ventilation with respiratory outcomes, adjusting for confounders (maternal smoking during pregnancy, gestational age, surfactant replacement therapy, and BMI).

**Results:**

No difference in lung function evaluation between ventilated and unventilated children were found. No association was also found between mechanical ventilation with lung function parameters.

**Conclusion:**

Mechanical ventilation for a short period at birth in preterm children was not associated with lung function impairment at 11 years of age in our study sample. It remains to define if ventilation may have a short-term effect on lung function, not evident at 11 years of age.

## Introduction

Invasive mechanical ventilation (IMV) is used to assist or replace spontaneous breathing *via* the placement of an endotracheal tube into the trachea through the patient’s mouth or nose. The endotracheal tube is connected to a device that delivers a definite amount of oxygen and volume of air, along with a set number of breaths per minute (1).

In 1967, Northway et al. (2) defined bronchopulmonary dysplasia (BPD) as lung injury with a need for supplemental oxygen at 28 days of postnatal age in preterm infants who required IMV for at least 1 week. BPD was characterized by severe lung injury with lung inflammation, peribronchial fibrosis, and pulmonary vascular smooth muscle hypertrophy.

Studies have investigated the short- and long-term effects of IMV at birth on lung function and structure. Detrimental effects on lung function have already been shown in the pre-surfactant era (3, 4).

During the last three decades, a protective lung approach characterized by gentler ventilation techniques, routine use of antenatal corticosteroids, and surfactant therapy has played a pivotal role in the reduction of neonatal mortality and morbidity (5–7). In addition, the improvement of the perinatal care decreased the role of IMV on lung consequences in ex-preterm children compared to the past (8). Nowadays, gentler ventilation techniques allow the survival of infants at lower gestational ages than in the past. Therefore, the type of lung injury is different compared to that originally described by Northway (9). “*Old*” BPD is caused by high oxygen concentrations and ventilation pressures. Differently, “*new*” BPD is due to the interaction among the following factors: interrupted alveolar and vascular development, ante- and postnatal injury, and lung reparative processes. It is characterized by histopathologic evidence of alveolar simplification with fewer and larger alveoli (10, 11).

A large amount of children born preterm still develops long-term respiratory complications (8). During the post-surfactant era, recent studies confirmed the presence of lung function impairment in children born prematurely at 11 years of age (12–14). In addition, the reduced gas diffusion in ex-preterm children at 7–11 years of age (15, 16) reflected the effect of extremely premature birth during an immature stage of lung development (17).

To date, IMV still remains a major cause of lung injury and BPD (7). The exclusive effect of premature birth and IMV on lung structural and functional injury is difficult to define. In addition, it is still unclear whether the effect of IMV may be exclusively short term for the action of reparative mechanisms (18) or long term affecting lung function trajectories in adulthood (19). Some authors stated that the severity of lung injury induced by IMV may depend on the duration of the insult (3, 4, 20).

Although there is growing literature on short- and long-term respiratory outcomes in infants born prematurely, there is still limited information on the subgroup of patients who require IMV in the post surfactant era. Hence, the aim of this study was to assess the effect of IMV in children born ≤ 32 weeks of gestational age on lung function parameters at 11 years of age.

## Materials and Methods

### Study Design and Population

**FIGURE 1:**
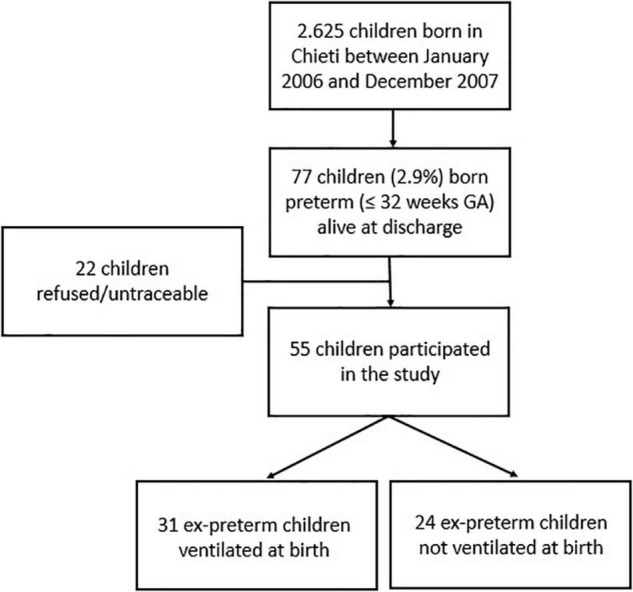
Flow chart of the study GA, gestational age.

The study was carried out at the Pediatric Allergy and Respiratory Unit of the University of Chieti. Children born in Chieti in 2006–2007 were 2,625. Infants born ≤ 32 weeks of gestational age and alive at discharge were 77 (2.9%); 55 ex-preterm children were enrolled in the study at 11 years of age, while 22 refused or were untraceable. Asthma and atopy were not exclusion criteria. The study was approved by the Ethical Committee of the University of Chieti (protocol number 4205), and written consent was obtained from the parents of the enrolled children. Figure 1 provides the flow chart of the study.

### Respiratory Health Outcomes and Covariates

Perinatal information was obtained by consulting medical records. At the follow-up visit (median age 11 years; 1–99% range 10–12.5 years), an accurate family and personal medical history were collected by a pediatric pulmonologist. The questionnaires included information about family history of asthma and allergy, smoking during pregnancy, child’s breastfeeding, passive smoking, pet keeping, preschool wheezing, ever asthma, and current asthma. Preschool wheezing was defined as physician-diagnosed wheezing from birth to 5 years of age. Current asthma was defined as physician-diagnosed asthma or asthma medication use in the past 12 months. Questions on wheezing and asthma were based on the International Study on Asthma and Allergy in Childhood (ISAAC) questionnaire (21).

The pediatric pulmonologist assessed anthropometric parameters (height, weight, BMI) and pubertal stage by a clinical evaluation. We created age- and sex-adjusted *z*-scores for BMI according to the Italian reference data (22).

The allergic evaluation was assessed by a skin prick test for the most common inhalant allergens (grass, house dust mite, cat and dog dander, mugwort, ragweed, molds). Histamine (10 mg/ml) and saline were considered positive and negative controls, respectively; diameters ≥ 3 mm were considered positive (23).

### Lung Function and Airway Inflammation Evaluation

At the visit, participants were in stable clinical condition without having experienced any respiratory disease in the previous 2 weeks. Lung function and lung volumes were measured by flow/volume curves and standardized body plethysmography according to ATS/ERS guidelines (24). The main parameters obtained were forced expiratory volume in the 1st second (FEV_1)_, forced vital capacity (FVC), FEV_1_/FVC ratio, forced expiratory flows between 25 and 75% of the FVC (FEF_75_, FEF_25–75_), total lung capacity (TLC), specific airways resistances (sRaw), and residual volume (RV). Lung function parameters were obtained at least 3 times for each participant; the maximally tolerated variability for the 3 evaluations was considered less than 10% (25).

Diffusing lung capacity test (DLCO) was measured by a standardized single breath technique (Vmax Autobox V62J, Carefusion, Hoechberg, Germany) according to ERS/ATS recommendations (26). Participants had no anemia.

We obtained *z*-scores of DLCO, FEV_1_, FVC, FEF_75_, FEF_25–75_, and FEV_1_/FVC using prediction equations from the Global Lung Initiative (GLI-2012) (27, 28) and specialized software (29). The lower limit of normal (LLN) was considered at the 5th percentile of the *z*-score distribution, (27) corresponded to –1.64. TLC, RV, and sRaw were expressed as the percentage of predicted values for age, height, sex, and ethnicity according to GLI-2012 reference values (28).

We measured fractional exhaled nitric oxide (FeNO) with an online method using a single breath exhalation and a sensitive chemiluminescence assay (Ecomedics CLD 88) according to ATS-ERS recommendations (30).

### Statistical Analysis

Continuous data were expressed as mean and SD or median and range 5–95%. Categorical data were presented as numbers and percentages. We compared the characteristics of ventilated ex-preterm children and unventilated ex-preterm children (controls) by using independent samples *T*-tests, Mann–Whitney U tests, and Pearson’s Chi-square tests.

A multivariable linear or logistic regression analysis was performed to examine the associations of IMV duration expressed as a continuous variable with respiratory outcomes taking possible confounders into account (maternal smoking during pregnancy, gestational age, surfactant replacement therapy, and BMI). Confounders were selected from literature first and were subsequently tested for their association with both the determinant and the outcome, or a change of the unadjusted effect estimates of 10% when added to the univariate model (31–34).

All measures of association are presented as odds ratios or *z*-scores differences and their corresponding 95% confidence intervals.

The statistical significance level was *p* < 0.05. SPSS version 25.0 for Windows (IBM, Armonk, NY, United States) and STATA/IC 15.1 (StataCorp. 2017. *Stata Statistical Software: Release 15*. StataCorp LLC. College Station, TX, United States) were used to perform the statistical analyses.

## Results

The study group was characterized by 31 ex-preterm children who needed IMV at birth and 24 unventilated ex preterm children. All participants were Caucasian. A diagnosis of BPD based on the oxygen needs for 28 days and additional oxygen or ventilation requirements at 36 weeks’ postmenstrual age was found in 5 (9.1%) of the study population. Social-demographic and clinical characteristics of the study population are shown in Table 1. No difference was found for all characteristics between ventilated and unventilated children, except for birth weight [1.3 (1.2–1.4) vs. 1.6 (1.4–1.7); *p* = 0.007], maternal smoking during pregnancy [0 (0%) vs. 7 (29.2%); *p* = 0.001], and surfactant administration [31 (100%) vs. 16 (66.7%); *p* = 0.001]. Surfactant was administered in all ventilated children and in 66.7% of unventilated ones through INSURE technique. Noteworthy, our preterm children were mechanically ventilated for a median period of 1.0 (0.5–17.0) days. Regarding lung function evaluation, there was no difference in all respiratory parameters between the two groups (Table 2).

**TABLE 1:** Birth and clinical characteristics of ex-preterm children.

	All	Ventilated children	Not ventilated children	*p*
Subjects *n* (%)	55	31 (56.4)	24 (43.6)	
Male sex (%)	27 (49.1)	14 (45.2)	13 (54.2)	0.508
Age (years)	11.0 (0.6)	10.9 (0.6)	11.2 (0.6)	0.976^#^
Weight (Kg)	42.3 (10.1)	42.0 (10.8)	42.7 (9.1)	0.596^#^
Height (cm)	145.3 (8.1)	144.8 (9.0)	145.8 (6.8)	0.672^#^
BMI (Kg/m^2^)	19.8 (3.4)	19.7 (3.5)	19.9 (3.3)	0.574^#^
**Birth characteristics**				
Gestational age at birth (weeks)	30.6 (30.2–31.1)	30.1 (29.5–30.7)	31.4 (30.9–31.9)	0.998^§^
< 28 weeks of gestation (%)	4 (7.3)	3 (9.7)	1 (4.2)	0.435
Birthweight (Kg)	1.4 (1.3–1.5)	1.3 (1.2–1.4)	1.6 (1.4–1.7)	**0.007** ^§^
Twins (%)	19 (34.6)	13 (42.0)	6 (25.0)	0.190
IMV (days)		1.0 (0.5–17.0)	0 (0.0–0.0)	**< 0.001** ^§^
CPAP (days)		4.0 (0.0–33.0)	2.0 (0.0–22.0)	0.864^§^
Oxygen-therapy (days)		6.0 (0.25–36.9)	1.5 (0.0–34.0)	0.817^§^
Surfactant (%)	47 (14.5)	31 (100)	16 (66.7)	**0.001**
**Maternal characteristics**				
Mother age (years)	31.3 (4.8)	32.3 (4.3)	30.2 (5.2)	0.053^#^
Cesarean section (%)	54 (98.1)	31 (100)	23 (95.8)	0.251
Smoking during pregnancy (%)	7	0 (0.0)	7 (29.2)	**0.001**
Maternal asthma (%)	8 (14.6)	7 (22.6)	1 (4.2)	0.055
*Comorbidities during pregnancy (%)	14 (25.4)	8 (25.8)	6 (25.0)	0.946
Family history of asthma (%)	11 (20.0)	8 (25.8)	3 (12.5)	0.221
Family history of inhalant allergy (%)	19 (34.5)	11 (35.5)	9 (37.5)	0.820
**Clinical characteristics**				
Bronchiolitis (%)	5 (9.1)	2 (6.5)	3 (12.5)	0.439
Pneumonia (%)	5 (9.1)	2 (6.5)	3 (12.5)	0.439
Preschool wheezing (%)	15 (27.3)	8 (25.8)	7 (29.2)	0.781
School-aged asthma (%)	6 (10.9)	3 (9.7)	3 (12.5)	0.739
Current asthma (%)	3 (5.4)	2 (6.5)	1 (4.2)	0.711
Ever asthma (%)	18 (32.7)	10 (32.3)	8 (33.3)	0.933
Positive SPT (%)	21 (61.8)	12 (38.7)	9 (37.5)	0.927
Eosinophilia	3.5 (2.4)	3.7 (2.6)	3.3 (2.4)	0.376

Data are presented as n, mean ± SD, n (%), or median (5–95% range). BMI, body mass index; IMV, invasive mechanical ventilation; CPAP, continuous positive airway pressure; SPT, skin prick test. *Comorbidities in pregnancy included gestational diabetes, risk of miscarriage, premature rupture of membranes, gestosis. Bold formatting to values where P-values is < 0.05. P-values from Pearson’s Chi-squared test. ^#^P-values from Unpaired t-Test. ^§^P-values from Main–Whitney U-test.

**TABLE 2:** Evaluation of respiratory parameters in ventilated and not ex-preterm children at 11 years of age.

	All	Ventilated children	Not ventilated children	*p*
Subjects *n* (%)	55 (100)	31 (56.4)	24 (43.6)	
**Respiratory parameters**				
FEV_1_ (*z*-score)	0.5 (1.3)	0.8 (1.2)	0.3 (0.2)	0.945^#^
FVC (*z*-score)	0.2 (1.2)	0.03 (1.3)	0.4 (1.1)	0.914^#^
FEV_1_/FVC (*z*-score)	0.6 (1.0)	0.6 (1.2)	0.6 (0.9)	0.902^#^
FEF_75_ (*z*-score)	1.2 (1.1)	1.0 (1.1)	1.3 (1.1)	0.915^#^
FEF_25–75_ (*z*-score)	0.3 (0.9)	0.2 (0.9)	0.4 (0.8)	0.827^#^
*positive bronchodilator response, *n* (%)	5 (9.1)	4 (12.9)	1 (4.2)	0.264^#^
TLC (% of the predicted value)	94.0 (79.0–141.0)	94.0 (81.0–141.0)	96.5 (79.0–141.0)	0.514^§^
RV (% of the predicted value)	99.0 (43.0–386.0)	100.0 (43.0–326.0)	93.5 (48.0–224.0)	0.592^§^
sRaw (% of the predicted value)	181.5 (109.0–355.0)	189.5 (117.0–354.0)	173.5 (109.0–355.0)	0.428^§^
DLCO (*z*-score)	–0.8 (1.2)	–0.5 (0.9)	–0.8 (1.2)	0.962^#^
FeNO (ppb)	9.1 (3.8–27.2)	9.1 (3.8–27.2)	11.3 (4.4–22.1)	0.461^§^

Data are presented as mean ± SD or median (5-95% range). FEV1, forced expiratory volume in 1 s; FVC, forced vital capacity; FEF_75_, forced expiratory flow at 75% of FVC; FEF_25–75_, forced expiratory flow at 25–75% of FVC; *post FEV1, FEV1 evaluated after giving a bronchodilator waiting 15 min. TLC, total lung capacity; RV, residual volume; sRaw, specific airway resistance; DLCO, diffusing capacity for carbon monoxide; FeNO, fractional exhaled nitric oxide; ppb, parts per billion. Bold formatting to values where p-value is < 0.05. P-values from Pearson’s Chi-squared test. ^#^P-values from Unpaired t-Test. ^§^P-values from Main–Whitney U-test.

In the crude model, no association was found of IMV with FEV_1_ *z*-score (β = –0.22, 95% CI: –1.23 to 0.13), FEF_75_ *z*-score (β = –0.13, 95% CI: –0.88 to 0.32), and FEF_25–75_ *z*-score (β = –0.13, 95% CI: –0.70 to 0.25). In addition, we found no association of IMV with specific respiratory resistance and lung volumes [sRaw% (β = –0.07, 95% CI: –20.27 to 33.82), VR% (β = –0.11, 95% CI: –18.92 to 41.81), and TLC% (β = –0.04, 95% CI: –9.53 to 7.13)]. Importantly, we observed no association of IMV with DLCO *z*-score (β = –0.25, 95% CI: –1.22 to 0.06). No association was found of IMV with eosinophilic inflammatory characteristics [FeNO (β = –0.11, 95% CI: –4.22 to 1.93) and peripheral eosinophilia (β = –0.10, 95% CI: –2.65 to 4.70)]. Additionally, no association was also found of IMV with all aforementioned respiratory parameters after adjustment for smoking during pregnancy, gestational age, surfactant replacement therapy, and BMI (Table 3).

**TABLE 3:** Association of mechanical ventilation at birth with respiratory parameters in ex-preterm children at 11 years of age.

		FEV_1_ *z*-score (95% CI)	FVC *z*-score (95% CI)	FEV_1_/FVC *z*-score (95% CI)	FEF_75_ *z*-score (95% CI)	FEF_25–75_ *z*-score (95% CI)	TLC% (95% CI)	VR% (95% CI)	sRaw% (95% CI)	DLCO *z*-score (95% CI)	FeNO (ppb) (95% CI)
Subjects	*n*	31	31	31	31	31	31	31	31	31	31
CRUDE MODEL *p*-value	31	–0.2 (1.2–1.3) *p* = 0.110	–0.2 (–1.1–0.2) *p* = 0.170	–0.02 (–0.6–0.5) *p* = 0.902	–0.1 (–0.9–0.3) *p* = 0.348	–0.1 (–0.7–0.3) *p* = 0.347	–0.04 (–9.5–7.1) *p* = 0.773	0.1 (–0.2–41.8) *p* = 0.453	0.07 (–20.3–33.8) *p* = 0.617	–0.3 (–1.2–0.1) *p* = 0.074	–0.1 (–4.2–1.9) *p* = 0.458
CONFOUNDER MODEL *p*-value	31	–0.1 (–1.1–0.6) *p* = 0.514	–0.04 (–0.9–0.7) *p* = 0.794	–0.1 (–0.9–0.5) *p* = 0.579	–0.02 (–0.8–0.7) *p* = 0.909	–0.1 (–0.7–0.5) *p* = 0.742	–0.00 (–10.6–10.5) *p* = 0.993	0.04 (–33.4–42.4) *p* = 0.811	0.3 (–5.6–57.9) *p* = 0.104	–0.1 (–1.0–0.5) *p* = 0.439	0.1 (–2.7–4.7) *p* = 0.578

*Data are presented as z-score derived from linear regression model. FEV1, forced expiratory volume in 1 s; FVC, forced vital capacity; FEF_75_, forced expiratory flow at 75% of FVC; FEF_25–75_, forced expiratory flow at 25–75% of FVC; TLC, total lung capacity; RV, residual volume; sRaw, specific airway resistance; DLCO, diffusing capacity for carbon monoxide; FeNO, fractional exhaled nitric oxide. Confounder model is adjusted for maternal smoking during pregnancy, gestational age, surfactant replacement therapy and BMI. Bold formatting to values where p-value is <0.05.*

Importantly, we showed the independent effect of both the gestational age on DLCO *z*-score (β = 0.35, 95% CI: 0.04 to 0.47) and maternal smoking during pregnancy on sRaw% (β = 0.41, 95% CI: 13.22 to 104.28).

## Discussion

Despite the great neonatal care improvement, survivors of prematurity continue showing lung function impairment in later life, especially those with BPD (35). IMV is often indispensable for very preterm newborns even if it may lead to chronic lung injury (36).

In literature, most of the studies about the potential lung injury due to IMV at birth were carried out in animals, and only few studies were performed in humans. In our study including 55 children born ≤ 32 weeks of gestational age, we found no association between IMV at birth and lung function at 11 years of age. Children previously ventilated showed no difference in spirometric parameters and DLCO values compared with unventilated children.

In contrast to our findings, animal studies suggested that IMV led to chronic lung injury disrupting the molecular networks responsible for normal alveolar-capillary membrane development (35, 37). In an animal model with 16 newborn lambs delivered prematurely and mechanically ventilated for 3–4 weeks, Bland et al. (36) investigated the effect of IMV on lungs through serial chest radiographs and postmortem examination. All lambs developed chronic lung injury. Interstitial lung edema, increased pulmonary arteriolar smooth muscle and elastin, decreased numbers of small pulmonary arteries and veins, and decreased capillary surface density in the distal lung were documented postmortem in ventilated lambs compared with lambs born at term. The authors concluded that these pulmonary circulation abnormalities could contribute to abnormal respiratory gas exchange (36). Similarly, Coalson et al. (38) found alveolar hypoplasia and a significantly reduced volume density of vascular endothelium in the lungs of preterm baboons mechanically ventilated for several weeks compared with term animals.

Regarding human studies, Laughon et al. (39) showed that IMV was a risk factor for pulmonary deterioration in 1,340 infants born between 2002 and 2004, after gestational age and birth weight. In an observational cohort study including 164 children born before 32 weeks of gestation and mechanically ventilated for a median period of 31 days, IMV was considered a risk factor for a reduced growth rate of lung development over childhood and adolescence, inducing possible implications for lung function trajectories into the adulthood. Specifically, a longer duration of IMV was associated with a lower increase in FEV_1_% than predicted and a greater decline in FEV_1_/FVC (19). A meta-analysis of 7 trials with 3,289 infants confirmed that avoiding IMV prevents volutrauma and therefore reduces direct lung injury and the subsequent lung inflammatory response, a crucial factor in BPD development (40). Furthermore, Simpson et al. (13) showed worse lung function (FEV1 *z*-score –0.72) in 163 preterm infants at 9–11 years of age compared with our study population (FEV1 *z*-score 0.8). However, lung function reported by Simpson et al. (13) was still in the normal range, given that the lower limit of normality for a *z*-score is a value of –1.64 (28).

The ventilator-induced lung injury (VILI) is caused by mechanical trauma and a “biotrauma” (41). The mechanical trauma is due to the over distention at high lung volumes and collapse/reopening of airway units at low lung volumes. The mechanical stretch caused a biotrauma, characterized by the release of mediators associated with the activation of the immune response (42, 43).

The short duration of IMV and consequently the small number of participants with BPD in our cohort could explain our contrasting findings, in addition to the different follow-up periods evaluated. Indeed, our patients were treated with IMV for a median period of 1 day, and only five patients developed BPD. Additionally, lung function improves with increasing gestational age and birth weight (13, 44). The short duration of IMV and the small number of participants with BPD could be explained by the relatively low number of extremely low gestational age newborns in our cohort. The median gestational age of our study population was 30.6 weeks, and only 4 children (7.3%) were born under 28 weeks of gestation. This characteristic could contribute to better lung function in our cohort compared with studies including more premature newborns (13, 14). Indeed, Simpson et al. (13) included children with a median gestational age of 28 weeks, and the cohort investigated by Fawke et al. (14) was composed of 37% of children under 25 weeks of gestational age.

In the pre-surfactant era, Kolobow et al. (3) showed that 48 h of IMV at a peak inspiratory pressure of 50 cm H_2_O caused lung injury with decreased pulmonary compliance and reduced blood oxygenation in 25 adult healthy sheep. In addition, Tsuno et al. (4) observed a detrimental effect on lung function in 27 young healthy sheep when IMV at a peak inspiratory pressure of 30 cm H_2_O was prolonged for up to 48 h. Specifically, functional residual capacity was measured every 4 h during 48 h of IMV; the authors found a progressive decline in functional residual capacity. Therefore, in 1998, Dreyfuss et al. (20) already suggested that the severity of VILI was greatly influenced by the duration of IMV, concluding that the deleterious effect of IMV could manifest after at least 2 days of treatment.

Regarding biotrauma related to ventilation, exposure to 1 h of IMV was not associated with changes in the levels of inflammatory mediators. Plasma levels of IL-6, tumor necrosis factor, IL-10, and IL-1 receptor antagonist remained low after 1 h of IMV in 39 adults with no previous lung disease (45).

Caironi et al. (46) selected 20 studies including five healthy mammalian species (sheep, pigs, rabbits, rats, mice) aggressively ventilated. The authors extrapolated data on morphometry, ventilator settings, respiratory function, and duration of IMV and calculated lung stress (transpulmonary pressure) and strain (end inspiratory lung inflation/lung resting volume ratio) for each animal group. The time to achieve preterminal VILI varied widely (18–2,784 min), and the duration of IMV was closely correlated with lung strain. This latter study underlies the importance of a different susceptibility of species to VILI, mostly due to the heterogeneous animal size, being the smaller species more prone to VILI (20).

Identifying the effect of IMV on lung function is challenging because other confounding treatments are required for the survival of participants. To better investigate the exclusive effect of IMV on very immature lungs, studies were carried out on sheep ventilated *in utero*. Allison et al. (47) investigated fetal sheep ventilated *in utero* for 1, 6, or 12 h at 110 days of gestation, at a stage of lung development that resembles that of very preterm human infants (26–28 weeks). Lung tissue was collected at 12 h after *in utero* ventilation and compared with those of the unventilated fetuses. The authors demonstrated VILI in these very immature lungs in the presence of normal nutrition, blood gases, and a sterile environment. O’Reilly et al. (48) considered fetal sheep (75% of normal gestation at term) mechanically ventilated *in utero* for 6 or 12 h, after which lung tissues were collected; another group was studied 7 days after 12 h ventilation. Remodeling of the bronchiolar epithelium and walls that lasts for at least 7 days was found in ventilated sheep compared with age-matched unventilated fetuses. Therefore, these two studies showed that injuries on lung parenchyma and bronchioles were evident after 6 or 12 h of IMV and persisted for 7 days (47, 48).

Similarly, another study found structural lung injury 1 day after IMV, and no evidence of lung injury was found 15 days after IMV. Eleven sheep were ventilated *in utero* for 2 h with an injurious IMV protocol at 125 days of gestation (85% of normal gestation). Six sheep were killed the day after (126 days of gestation) and 5 sheep were killed after 15 days (140 days of gestation); eight sheep were unventilated. Lungs displayed signs of injury 1 day after IMV, but no evidence of injury was found 15 days after IMV. The authors stated that the immature ovine lung could spontaneously repair itself following a brief episode of injurious IMV without other concomitant treatment or intervention (18). This latter study highlights the potential short-term effect of IMV on lungs and consequently on lung function in later life. The short-term effect of IMV on the lungs could explain the absence of association of IMV with lung function parameters at 11 years of age.

We would suggest that duration of IMV above a certain threshold may cause a lung injury influencing lung function later in life. The longer duration of IMV in other studies could explain the lung function alterations found. For instance, Simpson et al. (13) found abnormal lung structure and function at 9–11 years of life in 163 preterm children (99 with BPD) ventilated for a median period of 4 days.

The main strength of our study is the comprehensive respiratory evaluation of participants through the simultaneous assessment of lung function, diffusing capacity, and airway eosinophilic inflammation. Second, we included preterm children reevaluated after a long follow-up period. In addition, we used appropriate statistical methods evaluating also the effect of several confounding factors. Lastly, lung function measurements were performed by the same operator with expertise in the field of lung disease.

However, several methodological limitations need to be discussed. First, the study design was a retrospective analysis without a lung function assessment in the first years of life. For this reason, we cannot evaluate the potential short-term effect of IMV and its changes over time. Nevertheless, the data on prematurity were obtained from medical records reducing the possibility of information bias. Second, the small sample size could have affected the power of the study not detecting the smaller differences between the two groups. Indeed, only differences > 0.8 SD can be demonstrated, and the possibility of smaller and clinically relevant differences cannot be excluded. Third, the possible selection of more healthy patients among the preterms could have led to a selection bias. Indeed, they were treated with IMV only for a median period of 1 day. Furthermore, although we adjusted for several confounders, it was difficult to investigate the exclusive contribution of IMV to VILI independently from the additional treatments required for the survival of preterm infants leading to residual confounders. Finally, 19 participants were twins, and the familial/genetic predisposition could affect the interpretation of study outcome (49).

## Conclusion

We found that IMV at birth in preterm children was not associated with lung function impairment at 11 years of age. We would suggest that IMV for a short period may have a short-term effect on lung function, not evident at 11 years of age. The reparative capacity over time could compensate for the potential lung damage, especially if the injurious stimulus is brief. In the post-surfactant era, a brief duration of IMV in preterm infants could play a marginal role in lung function impairment later in life. Further longitudinal studies with a larger sample of preterm children who underwent a longer IMV and with lung function evaluation at different time points are needed to better characterize the type of effect of IMV (short vs. long) on lung function in the post-surfactant era. However, in clinical practice, our findings highlight the importance of reducing the IMV duration as much as possible and promoting alternative strategies to minimize the potential effects on lung function.

## Data Availability Statement

The raw data supporting the conclusions of this article will be made available by the authors, without undue reservation.

## Ethics Statement

The studies involving human participants were reviewed and approved by the Ethic Committee of University of Chieti. Written informed consent to participate in this study was provided by the participants or their legal guardian/next of kin.

## Author Contributions

PD: enrolment, original draft preparation, and writing. GD: writing and tables creation. SD and FC: supervision. MA: statistical analysis, supervision, and review. All authors have read and agreed to the published version of the manuscript.

## Conflict of Interest

The authors declare that the research was conducted in the absence of any commercial or financial relationships that could be construed as a potential conflict of interest.

## Publisher’s Note

All claims expressed in this article are solely those of the authors and do not necessarily represent those of their affiliated organizations, or those of the publisher, the editors and the reviewers. Any product that may be evaluated in this article, or claim that may be made by its manufacturer, is not guaranteed or endorsed by the publisher.

## Abbreviations

IMV: invasive mechanical ventilation
BPD: bronchopulmonary dysplasia
FEV1: forced expiratory volume in 1 s
FVC: forced vital capacity
FEF_75_: forced expiratory flow at 75% of FVC
FEF_25–75_: forced expiratory flow at 25–75% of FVC
TLC: total lung capacity
RV: residual volume
sRaw: specific airway resistance
DLCO: diffusing capacity for carbon monoxide
FeNO: fractional exhaled nitric oxide
CPAP: continuous positive airway pressure.

## References

[1] Walter K. Mechanical ventilation. *JAMA.* (2021) 326:1452. 10.1001/jama.2021.13084 34636861

[2] Northway WH Rosan RC Porter DY. Pulmonary disease following respiratory therapy of hyaline-membrane disease. Bronchopulmonary dysplasia. *N Engl J Med.* (1967) 276:357–68. 10.1056/NEJM196702162760701 5334613

[3] Kolobow T Moretti MP Fumagalli R Mascheroni D Prato P Chen V Severe impairment in lung function induced by high peak airway pressure during mechanical ventilation. *Am Rev Respir Dis.* (1987) 135:312–5. 3544984 10.1164/arrd.1987.135.2.312

[4] Tsuno K Prato P Kolobow T. Acute lung injury from mechanical ventilation at moderately high airway pressures. *J App Physiol.* (1990) 69:956–61. 10.1152/jappl.1990.69.3.956 2123181

[5] Ronkainen E Dunder T Peltoniemi O Kaukola T Marttila R Hallman M. New BPD predicts lung function at school age: follow-up study and meta-analysis. *Pediatr Pulmonol.* (2015) 50:1090–8. 10.1002/ppul.23153 25589379

[6] Jobe AH Ikegami M. Lung development and function in preterm infants in the surfactant treatment era. *Annu Rev Physiol.* (2000) 62:825–46. 10.1146/annurev.physiol.62.1.825 10845113

[7] Brown MK DiBlasi RM. Mechanical ventilation of the premature neonate. *Respiratory Care.* (2011) 56:1298–313. 21944682 10.4187/respcare.01429

[8] Priante E Moschino L Mardegan V Salvadori S Baraldi E. Respiratory outcome after preterm birth: a long and difficult journey. *Am J Perinatol.* (2016) 33:1040–2. 10.1055/s-0036-1586172 27603531

[9] Mahut B De Blic J Emond S Benoist MR Jarreau PH Lacaze-Masmonteil T Chest computed tomography findings in bronchopulmonary dysplasia and correlation with lung function. *Arch Dis Child Fetal Neonatal Ed.* (2007) 92:F459–64. 10.1136/adc.2006.111765 17379740 PMC2675392

[10] Bancalari E Jain D. Bronchopulmonary dysplasia: 50 years after the original description. *Neonatology.* (2019) 115:384–91. 10.1159/000497422 30974430

[11] Baker CD Alvira CM. Disrupted lung development and bronchopulmonary dysplasia: opportunities for lung repair and regeneration. *Curr Opin Pediatr.* (2014) 26:306–14. 10.1097/MOP.0000000000000095 24739494 PMC4121955

[12] Kaplan E Bar-Yishay E Prais D Klinger G Mei-Zahav M Mussaffi H Encouraging pulmonary outcome for surviving, neurologically intact, extremely premature infants in the postsurfactant era. *Chest.* (2012) 142:725–33. 10.1378/chest.11-1562 22423043

[13] Simpson SJ Logie KM O’Dea CA Banton GL Murray C Wilson AC Altered lung structure and function in mid-childhood survivors of very preterm birth. *Thorax.* (2017) 72:702–11. 10.1136/thoraxjnl-2016-208985 28119488

[14] Fawke J Lum S Kirkby J Hennessy E Marlow N Rowell V Lung function and respiratory symptoms at 11 years in children born extremely premature. The EPICure study. *Am J Respir Crit Care Med.* (2010) 182:237–45. 10.1164/rccm.200912-1806OC 20378729 PMC2913237

[15] Di Filippo P Giannini C Attanasi M Dodi G Scaparrotta A Petrosino MI Pulmonary outcomes in children born extremely and very preterm at 11 years of age. *Front Pediatr.* (2021) 9:635503. 10.3389/fped.2021.635503 34113584 PMC8185052

[16] Cazzato S Ridolfi L Bernardi F Faldella G Bertelli L. Lung function outcome at school age in very low birth weight children. *Pediatr Pulmonol.* (2013) 48:830–7. 10.1002/ppul.22676 23129340

[17] De Paepe ME Mao Q Powell J Rubin SE DeKoninck P Appel N Growth of pulmonary microvasculature in ventilated preterm infants. *Am J Respir Crit Care Med.* (2006) 173:204–11. 10.1164/rccm.200506-927OC 16210670 PMC2662989

[18] Brew N Hooper SB Allison BJ Wallace MJ Harding R. Injury and repair in the very immature lung following brief mechanical ventilation. *Am J Physiol Lung Cell Mol Physiol.* (2011) 301:917–26. 10.1152/ajplung.00207.2011 21890511

[19] Levin JC Sheils CA Gaffin JM Hersh CP Rhein LM Hayden LP. Lung function trajectories in children with post prematurity respiratory disease: identifying risk factors for abnormal growth. *Respir Res.* (2021) 22:143. 10.1186/s12931-021-01720-0 33971884 PMC8112031

[20] Dreyfuss D Saumon G. Ventilator-induced lung injury. *Am J Respir Crit Care Med.* (1998) 157:294–323. 10.1164/ajrccm.157.1.9604014 9445314

[21] Asher MI Keil U Anderson HR Beasley R Crane J Martinez F International study of asthma and allergies in childhood (ISAAC): rationale and methods. *Eur Res J.* (1995) 8:483–91. 10.1183/09031936.95.08030483 7789502

[22] Cacciari E Milani S Balsamo A Spada E Bona G Cavallo L Italian cross-sectional growth charts for height, weight and BMI (2 to 20 yr). *J Endocrinol Invest.* (2006) 29:581–93. 10.1007/BF03344156 16957405

[23] van Kampen V de Blay F Folletti I Kobierski P Moscato G Olivieri M EAACI position paper: skin prick testing in the diagnosis of occupational type I allergies. *Allergy.* (2013) 68:580–4. 10.1111/all.12120 23409759

[24] Miller MR Hankinson J Brusasco V Burgos F Casaburi R Coates A Standardisation of spirometry. *Eur Respir J.* (2005) 26:319–38. 10.1183/09031936.05.00034805 16055882

[25] Pellegrino R Viegi G Brusasco V Crapo RO Burgos F Casaburi R Interpretative strategies for lung function tests. *Eur Respir J.* (2005) 26:948–68. 10.1183/09031936.05.00035205 16264058

[26] Graham BL Brusasco V Burgos F Cooper BG Jensen R Kendrick A 2017 ERS/ATS standards for single-breath carbon monoxide uptake in the lung. *Eur Respir J.* (2017) 49:1600016. 10.1183/13993003.00016-2016 28049168

[27] Stanojevic S Graham BL Cooper BG Thompson BR Carter KW Francis RW Official ERS technical standards: global lung function initiative reference values for the carbon monoxide transfer factor for caucasians. *Eur Respir J.* (2017) 50:1700010. 10.1183/13993003.00010-2017 28893868

[28] Quanjer PH Stanojevic S Cole TJ Baur X Hall GL Culver BH Multi-ethnic reference values for spirometry for the 3-95-yr age range: the global lung function 2012 equations. *Eur Respir J.* (2012) 40:1324–43. 10.1183/09031936.00080312 22743675 PMC3786581

[29] Quanjer PH Stanojevic S Cole TJ Baur X Hall GL Culver BH *GLI-2012 data Conversion Software.* (2012). Available online at: www.lungfunction.org/tools.html (accessed November 7, 2021).

[30] American Thoracic Society, European Respiratory Society. ATS/ERS recommendations for standardized procedures for the online and offline measurement of exhaled lower respiratory nitric oxide and nasal nitric oxide, 2005. *Am J Respir Crit Care Med.* (2005) 171:912–30. 10.1164/rccm.200406-710ST 15817806

[31] Lista G Castoldi F Bianchi S Lupo E Cavigioli F Farolfi A Lung function and respiratory health at school age in ventilated very low birth weight infants. *Indian J Pediatr.* (2014) 81:275–8. 23868538 10.1007/s12098-013-1129-1

[32] Doyle LW Andersson S Bush A Cheong JLY Clemm H Evensen KAI Expiratory airflow in late adolescence and early adulthood in individuals born very preterm or with very low birthweight compared with controls born at term or with normal birthweight: a meta-analysis of individual participant data. *Lancet Respir Med.* (2019) 7:677–86. 10.1016/S2213-2600(18)30530-7 31078498

[33] Gunlemez A Er I Baydemir C Arisoy A. Effects of passive smoking on lung function tests in preschool children born late-preterm: a preventable health priority. *J Matern Fetal Neonatal Med.* (2019) 32:2412–7. 10.1080/14767058.2018.1430759 29353510

[34] Di Filippo P Lizzi M Raso M Di Pillo M Chiarelli F Attanasi M. The role of breastfeeding on respiratory outcomes later in childhood. *Front Pediatr.* (2022) 10:829414. 10.3389/fped.2022.829414 35573946 PMC9096137

[35] Ahlfeld SK Conway SJ. Assessment of inhibited alveolar-capillary membrane structural development and function in bronchopulmonary dysplasia. *Birth Defects Res A Clin Mol Teratol.* (2014) 100:168–79. 10.1002/bdra.23226 24604816 PMC3999962

[36] Bland RD Albertine KH Carlton DP Kullama L Davis P Cho SC Chronic lung injury in preterm lambs: abnormalities of the pulmonary circulation and lung fluid balance. *Pediatr Res.* (2000) 48:64–74. 10.1203/00006450-200007000-00013 10879802

[37] Coalson JJ Winter V deLemos RA. Decreased alveolarization in baboon survivors with bronchopulmonary dysplasia. *Am J Resp Care.* (1995) 152:640–6. 10.1164/ajrccm.152.2.7633720 7633720

[38] Coalson JJ Winter VT Siler-Khodr T Yoder BA. Neonatal chronic lung disease in extremely immature baboons. *Am J Respir Crit Care Med.* (1999) 160:1333–46. 10508826 10.1164/ajrccm.160.4.9810071

[39] Laughon M Allred EN Bose C O’Shea TM Van Marter LJ Ehrenkranz RA Patterns of respiratory disease during the first 2 postnatal weeks in extremely premature infants. *Pediatrics.* (2009) 123:1124–31. 19336371 10.1542/peds.2008-0862 PMC2852187

[40] Fischer HS Buhrer C. Avoiding endotracheal ventilation to prevent bronchopulmonary dysplasia: a meta-analysis. *Pediatrics.* (2013) 132:1351–60. 10.1542/peds.2013-1880 24144716

[41] Thekkeveedu RK Guaman MC Shivanna B. Bronchopulmonary dysplasia: a review of pathogenesis and pathophysiology. *Respir Med.* (2017) 132:170–7. 10.1016/j.rmed.2017.10.014 29229093 PMC5729938

[42] Curley GF Laffey JG Zhang H Slutsky AS. Biotrauma and ventilator-induced lung injury. *Chest.* (2016) 150:1109–17. 27477213 10.1016/j.chest.2016.07.019

[43] Zhang H Zhang J Zhao S. Airway damage of prematurity: the impact of prolonged intubation, ventilation, and chronic lung disease. *Semin Fetal Neonatal Med.* (2016) 21:246–53. 10.1016/j.siny.2016.04.001 27129915

[44] Di Filippo P Dodi G Ciarelli F Di Pillo S Chiarelli F Attanasi M. Lifelong lung sequelae of prematurity. *Int J Environ Res Public Health.* (2022) 19:5273. 10.3390/ijerph19095273 PMC9104309 35564667

[45] Wrigge H Zinserling J Stüber F von Spiegel T Hering R Wetegrove S Effects of mechanical ventilation on release of cytokines into systemic circulation in patients with normal. *Anesthesiology.* (2000) 93:1413–7. 11149435 10.1097/00000542-200012000-00012

[46] Caironi P Langer T Carlesso E Protti A Gattinoni L. Time to generate ventilator-induced lung injury among mammals with healthy lungs?: a unifying hypothesis. *Intensive Care Med.* (2011) 37:1913–20. 10.1007/s00134-011-2388-9 22052185

[47] Allison BJ Crossley KJ Flecknoe SJ Davis PG Morley CJ Harding R Ventilation of the very immature lung in utero induces injury and BPD-like changes in lung structure in fetal sheep. *Pediatr Res.* (2008) 64:387–92. 10.1203/PDR.0b013e318181e05e 18552709

[48] O’Reilly M Hooper SB Allison BJ Flecknoe SJ Snibson K Harding R Persistent bronchiolar remodeling following brief ventilation of the very immature ovine lung. *Am J Physiol Lung Cell Mol Physiol.* (2009) 297:992–100. 10.1152/ajplung.00099.2009 19717553

[49] Hibbs AM Black D Palermo L Cnaan A Luan X Truog WE Accounting for multiple births in neonatal and perinatal trials: systematic review and case study. *J Pediatrics.* (2010) 156:202–8. 10.1016/j.jpeds.2009.08.049 19969305 PMC2844328

